# “They Handcuffed Him, Shackled Him, all of that—in Front of Her”: Family Experiences and Considerations Upon Arrest of a Parent

**DOI:** 10.1007/s40653-025-00696-z

**Published:** 2025-03-04

**Authors:** Jordan F. Pollard, Leslie N. Jones, Elizabeth G. Keller, Ebony Ruhland, Kelly J. Kelleher, Deena J. Chisolm, Samantha J. Boch

**Affiliations:** 1 School Psychology, University of Cincinnati, Cincinnati, OH USA; 2 Abigail Wexner Research Institute at Nationwide Children’S Hospital, Columbus, OH USA; 3 UCLA Fielding School of Public Health, Los Angeles, CA USA; 4 College of Nursing, University of Cincinnati, Cincinnati, OH USA; 5 School of Criminal Justice, Rutgers University, Newark, NJ USA; 6 Department of Pediatrics, The Ohio State University College of Medicine, Columbus, OH USA; 7 James M. Anderson Center for Health Systems Excellence, Cincinnati Children’s Hospital Medical Center, Cincinnati, OH USA

**Keywords:** Parental incarceration, Children and youth, Adverse childhood experiences, Prison, Jail

## Abstract

Since 1980 in the United States (US), more than 10 million arrests have occurred each year. With a majority of those incarcerated being parents, millions of children and remaining household members are adversely affected. Despite the volume of US arrests, few studies provide family context and child considerations about the time period of arrest. This study sought to describe family experiences and considerations to best support youth during parental arrest. Qualitative data were gathered using semi-structured, in-depth phone interviews from March to August 2020 with adolescents (12–18 years) who have had a parent incarcerated, caregivers of children of incarcerated parents, and parents upon one year of release of incarceration. Participants were recruited using flyers and emails to community-based organizations and schools. General themes emerged through qualitative content analysis and inductive open coding procedures. Data from 26 participants were summarized (10 adolescents, 10 caregivers, and six parents upon one year of release from jail/prison). Upon arrest, family experiences were described as traumatizing and stigmatizing regardless of whether the child was present to observe the arrest. The effects of witnessing the arrest were influenced by officer treatment. Families shared how limited household- and community-based resources were available to help the family cope with the consequences of the arrest. Results underscore the need for family-centered approaches and improved interventions upon arrest that may better support children and family members during this time. Recommendations for better transparency, connection, and transition supports are discussed.

## Introduction

The United States (US) maintains the largest criminal legal system in the world, incarcerating the most people per capita, for several decades (Walmley, 2018). In addition to large size, the US correctional system has incredible churn. Since 1980, over 10.5 million arrests have occurred annually (or on average, around 30,000 daily arrests) (Vera, n.d). Unfortunately, most of the adults behind bars are parents, which can spur serious social, economic, academic, and household consequences for their children and family members. Nearly one in every 14 US youth have had a parent incarcerated (Murray et al., 2012), and nearly half of children with an incarcerated parent are younger than nine years old (Glaze & Maruschak, 2016). Due to centuries of racially discriminative policing and sentencing, families of color are disproportionately affected by parental arrest and incarceration. About one in every nine Black youth, compared to one in every 17 White youth have had an incarcerated parent in jail or prison (Boch & Ford, 2021).

The impact of parental incarceration (PI) on children and their familial environments is recognized in the US as an adverse childhood experience with two decades of well-documented effects on child health and development (Felitti, et al., 1998; Genty, 2002; Arditti, 2012; Giordano et al., 2019; Wildeman et al., 2019). Children with PI are more likely to experience adverse mental health outcomes (Lee & Wildeman, 2021; Murray et al., 2012; Testa & Jackson, 2020; Turney, 2014) and are at higher risk to become incarcerated when compared to children who are unexposed (Cox, 2009). Having a parent incarcerated has also been associated with additional childhood adversity (Boch et al., 2019) and poorer academic outcomes (Testa & Jackson, 2020; Turney, 2014).

PI has numerous psychological and economic effects on the family, household, and community (Arditti, 2005). At the center of the microsystem are family relationships, and research shows the significance of maintaining strong family connections especially when a parent is incarcerated and that connection is desired (Song et al., 2018). The primary way a family can remain connected during PI is via phone calls, letters, and visitations (Smith & Young, 2017). However, phone calls can be costly ($6 for about 15 min) (Wagner & Jones, 2019). Sometimes parents are incarcerated over 100 miles away, creating additional strain on family members due to the need for reliable transportation. As a result, families with parents incarcerated are burdened by the substantial cost of sustaining regular contact (Travis et al., 2016).At the macrosystem level, children and families must deal with the saliency of the stigmatization of incarceration. Unlike losing a family member to death or illness, losing a parent to incarceration rarely receives community sympathy (Arditti, 2012). Losing a parent to incarceration is a unique experience because the grieving process is often unresolved and confusing, due to the limited information being shared and delayed opportunity to grieve fully. Additionally, due the disproportionate rates of incarceration, the stigmatizing impact is heightened for children of color, those who reside in rural areas, and families of low-income backgrounds (Murphey & Cooper, 2015a, 2015b).

The specific incidents that create such negative effects have not been elucidated. Most of the literature on children of incarcerated parents does not differentiate between the time periods of incarceration (arrest, sentencing, detainment, release Johnson & Easterling, 2015; Keller et al., 2022) and potential impact. Recent research suggests that it is not uncommon for children to witness their parent’s arrest (Metcalfe et al., 2022). Of a sample of 1,869 children from the National Survey of Child and Adolescent Well-being, **nearly 40%** of these children had witnessed the arrest of a household member (Phillips & Zhao, 2010). Furthermore, a recent study of children 2 to 6 years old found that 27% of them witnessed their father’s arrest, 29% witnessed their father’s crime, and 15% witnessed both the arrest and the crime (Muentner et al., 2021). According to Phillips and Zhao (2010), witnessing a household member being arrested is predictive of posttraumatic symptoms among children. Children who experience an arrest of a parent are often left needing safe places to live and people to care for them, in addition to coping with the emotional toll of parental separation (San Francisco Partnership for Incarcerated Parents, n.d.). While arrest procedures by officers should prioritize the safety of the public in removing an adult from the home as quickly and as safely as possible, little is known about family experiences during arresting procedures. Additional qualitative research is warranted to provide further context and insight into the experiences of parental arrest from families’ own perspectives and voices.

### Objectives

This study sought to describe family experiences and considerations to best support youth during parental arrest. We include perspectives from adolescents, caregivers, and previously incarcerated parents within one year of release who have been part of the PI experience. In particular, we hoped that participants would identify specific needs, services and policies that might provide support to youth during the time of parental arrest.

## Methods

### Sampling and Recruitment

The study used a convenience-based sampling strategy via e-mail and flyer recruitment. We recruited the following three groups: 1) adolescents between the ages of 12–18 years who have experienced a parent’s incarceration; 2) parents (over the age of 18 years) who have been released from jail or prison within the last year; or 3) caregivers (over the age of 18 years) of children who have had incarcerated parents. We required that participants be English speaking and live in the US. Emails containing information about the study purpose, design, eligibility criteria, and recruitment flyers (for the organization to use at their discretion) were sent to numerous schools, reentry/transitional housing programs, and other community organizations that provide services to families that are affected by household incarceration primarily located within XXXX. Several persons shared our recruitment emails to other listservs including The National Resource Center on Children and Families of the Incarcerated, the Correctional Education Association of Wisconsin, Higher Education in Prison listserv, and the Wings for LIFE International listserv.

Semi-structured telephone interviews explored family experiences and recommendations to improve support for children of incarcerated parents. After a series of demographic questions, participants were asked questions about their experiences by the phase of incarceration (i.e., upon arrest, during incarceration, and upon release). Results corresponding with the upon release/or reintegration back home from incarceration (Keller et al., 2022) and the results regarding visitation during incarceration (Jones et al., 2023) are published elsewhere. The study questionnaire was informed by Bronfenbrenner’s Bioecological Model of Human Development (Bronfenbrenner, 1977) to capture family experiences across multiple contexts such as in the home, workplace, school, and communities. All participants provided verbal consent before participation. Participants were also instructed not to use the names, or any identifiable information of the parent incarcerated and/or youth. The study was approved by the XXXXXX Institutional Review Board.

### Data Collection and Analysis

Phone interviews were conducted from March 2020 to August 2020 by one trained research staff member. All interviews were recorded and transcribed. Incentive payments of $40 were given to interviewees in thanks for their time. The staff member who was responsible for recruitment, scheduling, and conducting the interviews indicated to the senior author that data saturation had been met due to consistent overlap in information after 26 interviews were completed. Qualitative content analysis occurred in two phases. The first phase occurred after the completion of transcription to determine initial codes. To do this, three research staff members independently reviewed each of the transcriptions and generated initial codes using open coding procedures to organize the data and create categories. In open coding procedures the researchers take raw qualitative data, break them down into smaller parts, and assign descriptive code (Strauss & Corbin, 2004). The researchers’ inductive approach was further guided by Bronfenbrenner’s ecological model, which informed the researchers’ a priori understanding of how PI impacts the family unit (Bronfenbrenner, 1977). For phase two of analysis, the research team met weekly for several months to compare, discuss, and modify codes based on a consensus for emerging patterns and themes by sub-group and eventually, by phase of incarceration. Broad themes were predominantly descriptive and became apparent after reviewing the codes and establishing patterns. Each stage of analysis involved individual then collective review as a research team until concordance of interpretation on codes, patterns, and themes were met. In the case of discrepancies in coding, validation of themes occurred through individual and team reviews which included two experts in health services research and pediatric health equity (coauthors KK and DC). More methodological detail is reported elsewhere (Keller et al., 2022). Direct quotes were selected individually by three research staff members. Then as a team, we confirmed that these quotes were representative of the experiences of the family.

## Results

The demographic characteristics of the adolescents and adults (caregivers and parents upon one year of release of incarceration) are summarized in Table 1. The final sample of participants (N = 26) consisted of 10 youths (38.5%), six parents upon one year of release from jail or prison (23.1%), and 10 caregivers of children with incarcerated parents (38.5%). Of the participating youths (n = 10), most identified as female (90.0%, n = 9) and 50.0% (n = 5) were older adolescents (16–17 years old). Forty percent (n = 4) of the adolescents identified as Black or African American. Four of the youth participants experienced a father’s incarceration, two experienced a mother’s incarceration, and four experienced both parents’ incarceration. Similar to the adolescent sample, most of the adult participants were female (75%, n = 12), and more than half identified as Black or African American (56.3% or n = 9). More than half of the adult participants were over the age of 45 years (75%, n = 12). Most of the caregivers of the children exposed to PI identified as grandparents (70%, n = 7). All participants lived in the US, with most residing in Ohio (50%, n = 13). In addition, three youth-caregiver familial dyads were among the participants. The total number of times a parent/parents were incarcerated, and the totaled sentence duration (or length of time spent incarcerated), varied widely among the youth, caregiver, and recently released parent sub-samples. About half of the youth and caregiver sample reported that one or both parents were sentenced for a total of nine years or longer (55.0%, n = 11 out of 20) and were incarcerated 5 or more times (45.0%, n = 9 out of 20). Parents released from jail or prison within the past year reported that they were incarcerated 1–2 times, and most indicated that their sentence length was five years or more (66.6%, n = 4 out of 6).

**Table 1:** Demographic and PI characteristics of the adolescent, caregiver, and previously incarcerated parents sample

Demographic Characteristics	Adolescent Sample	Caregiver Sample	Previously Incarcerated Parents Sample
	N = 10 % (n)	N = 10 % (n)	N = 6 % (n)
**Gender**			
Female	90.0% (9)	20% (10)	33.3% (2)
Male	10.0% (1)	0	66.6% (4)
**Race**			
Black or African American (AA)	40.0% (4)	60.0% (6)	50.0% (3)
White	30.0% (3)	30.0% (3)	33.3% (2)
Black or AA and White	20.0% (2)	–	–
American Indian or Native Alaskan (AI or NA) Other	0 10.0%(1)	– –	16.7% (1)
AI or NA and Other	–	1.0% (1)	–
**Ethnicity**			
Hispanic or Latino	0	10.0% (1)	–
**Age**			
Average (years) and Range	14.7 years (12–16)	47.6 years (28–67)	51.5 years (46–60)
12–13 years	30.0% (3)	–	–
14–15 years	20.0% (2)	–	–
16–17 years	50.0% (5)	–	–
25–34 years	–	20.0% (2)	–
35–44 years	–	20.0% (2)	–
45–54 years	–	30.0% (3)	66.6% (4)
> 55–64 years	–	30.0% (3)	33.3% (2)
**Living Situation**			
Mother and/or Father	80.0% (8)	–	–
Legal Guardian/Caregiver	20.0% (2)	–	–
**Type of School**			–
Charter or Private	40.0% (4)	–	–
Public	60.0% (6)	–	–
**Gender of Parent Incarcerated**			
Mother	20.0% (2)	10.0% (1)	–
Father	40.0% (4)	60.0% (6)	–
Both	40.0% (4)	30.0% (3)	–
**Current Grade or Highest Level of School**			
6th-7th grade	30.0% (3)	–	–
8th-9th grade	10.0% (1)	–	–
10th-11th grade	60.0% (6)	–	–
Some high school	–	–	16.7% (1)
High School Diploma or GED	–	10.0% (1)	16.7% (1)
Some college or 2 year degree	–	60.0% (6)	50% (3)
4 year college degree or higher	–	30.0% (3)	33.3% (2)
**Annual Household Income**	–		
Less than $15,000	–	10.0% (1)	33.3% (2)
$15,000 to $24,999	–	10.0% (1)	–
$25,000 to $49,999	–	50.0% (5)	33.3% (2)
$50,000 to $74,999	–	10.0% (1)	–
$75,000 to $99,999	–	–	–
$100,000 or more	–	–	16.7% (1)
Prefer not to answer		20.0% (2)	16.7% (1)
**Incarceration Context**^**a**^			
**Total Times/Incarcerations**			
Mother			Self-Incarceration History
1–2 incarcerations	10.0% (1)	–	33.3% (2)
3–5 incarcerations	10.0% (1)	–	–
> 5 incarcerations	–	10.0% (1)	–
Father			
1–2 incarcerations	30.0% (3)	20.0% (2)	66.6% (4)
3–5 incarcerations	–	20.0% (2)	–
> 5 incarcerations	10.0% (1)	20.0% (2)	–
Both Mother and Father			
1–2 incarcerations	–	10.0% (1)	–
3–5 incarcerations	10.0% (1)	–	–
> 5 incarcerations	30.0% (3)	20.0% (2)	–
**Total Sentence Length**			
Mother			
3 months- 1 year	10.0% (1)	–	–
1–4 years	–	10.0% (1)	–
5–8 years	–	–	33.3% (2)
> 9 years	10.0% (1)	–	–
Father			
3 months- 1 year	20.0% (2)	10.0% (1)	–
1–4 years	–	10.0% (1)	16.7% (1)
5–8 years	–	20.0% (2)	–
> 9 years	20.0% (2)	20.0% (2)	33.3% (2)
Prefer not to answer			16.7% (1)
Both Mother and Father			–
3 months- 1 year	–	–	–
1–4 years	–	–	–
5–8 years	–	10.0% (1)	–
> 9 years	40.0% (4)	20.0% (2)	–

^a^ Numbers were based on participants who responded. Incarceration included both exposures to jail and prison, but these were not distinguished as 6/10 youth use “jail/prison” interchangeably

The emergent themes included (1) effects of witnessing the arrest influenced by officer treatment, (2) trauma caused by concealing the arrest, and (3) limited household and community resources to help family deal with arrests. Please find Table 2 presents a summary of results.

**Table 2:** Summary of results

Themes	Family Centered Suggestions
Effects of Witnessing the Arrest Influenced by Officer Treatment	• Extra person to assist the officer during the arrest period • Standardization in arresting procedures
Trauma Caused by Concealing the Arrest	• Access to honest information about the arrest • Mental health supports to mitigate stigma and fear
Limited Household and Community Resources to Help Family Deal with Arrests	• Increasing legal supports for the incarcerated parent (i.e., ensuring parents have opportunities for housing, work, and basic needs) • Ensuring clear legal processes of accessing government support and custody of the child after the parent was detained • Standardizing legal assistance across counties • Increasing socioemotional support in the community

### Effects of Witnessing the Arrest Influenced by Officer Treatment

Adolescent experiences upon arrest varied on whether they or the caregivers were present to witness the arrest and how the families were treated by the arresting officer. About a quarter of all participants stated that a child witnessed the arrest of their parent (2 of the adolescent participants noted being present, and 5 of the adult participants noted that children were present). One adolescent expressed the emotions and fear of witnessing multiple parental arrests,

“So, for my dad, there [have] been a few times he was arrested, and I have been around. Both times, it was really, really scary and nerve racking because oh, like, ‘What’s going on, what’s happening?’ At the time I was really young for those times and then all the other times it was like that feeling of ‘what’s going on? Am I going to see my dad again?”

Adolescents who had a comforting officer or one who allowed the child to hug their parent, had a better reflection than those who did not. One adolescent shared that they had a comforting officer during the time of arrest,

“…[My dad]… came and picked me up… And we stopped at the Speedway…. A cop had pulled in the parking lot to Speedway…[my dad] threw the drugs he had down and … come to find out [my dad] had drugs all in the back seat, in the trunk, and the car we was in, was stolen… Then, they took me, and they put me in the cop car. But they knew I was his daughter and I didn’t have nothing to do with it... Then my mom came and got me. But yeah, that’s basically what… happened a month ago, that’s the most recent time I can think of that I was with him and I was present in the situation… I can say the cops were comforting me good, my mom came and got me. She took me out because she knew that was rough for me.”

Another shared, the importance of physical touch and the ability to say goodbye. A caregiver shared their daughter’s experience when their father was arrested, highlighting the importance of allowing the child to hug their parent upon arrest,

"… Six officers came into my house. And it was terrible, [my daughter] was completely traumatized, they wouldn’t let her near her father. They handcuffed him, shackled him, all of that - in front of her. She was screaming and crying, and they wouldn’t let her near him. And finally, one of the officers… was like, “Let that girl come over here and see her father and hug him”... So, he uncuffed her father, and let her go to her father, and let her father hug her and all of that, and definitely calmed her down before they took him out.”

Another caregiver described a similar experience with her grandson,

“[The detectives] did give [the arrested father] a chance to say goodbye to his son … and then I brought my grandson back…[to] his dad … [the detectives then] said, ‘We have some questions and some business to handle with your dad. And he’s going to come with us to [name of city where jail was located] … if you want to [say] goodbye to your dad. I don’t know how long he is going to be gone… I don’t know how long we are going to need his help, but we need his help with something…[they] hugged, and stuff and it was a little emotional because my son was a little emotional.

However, one caregiver noted disrespect during an arrest,

“Having an officer continue to repeat… [that the parent under arrest is] a piece of ****, or they break the law, you know to have them continue to repeat that was the least helpful thing. It didn’t make the situation any better or the impact of it.”

One grandmother caregiver with her own incarceration experience underscored the importance of the officers’ behavior during arrest,

“…I think the most helpful thing is the officers. It all starts with them. The way they come into a house and the way they treat this person that they are getting ready to arrest, forgetting that this is a father or a mother and that these children don’t see a criminal they see their parents. So, keeping that in mind when they are coming into a house and arresting somebody that they are looking at this person through one eyes, but [everyone] in that house is looking with a completely different set of eyes.

”Others offered how an extra person could assist the officer during the arrest period:

“I really feel strongly about the fact that they need to have, maybe not a police officer, but somebody that is assisting the officer that can comfort the child because the [officer’s] main reason is to you know take care of the bad guy. And at this point, my son was the bad guy and his child is… just stuck wondering …[and pleading,] “don’t take my dad”. To her, [her] dad is very good you know.”

### Trauma Caused by Concealing the Arrest

While witnessing an arrest can be emotionally challenging for youth, not witnessing the arrest can also create confusion. Adolescents who did not directly witness the arrest shared how receiving information regarding the details of a parent’s arrest or finding out about it later also created stress and pain. One adolescent who did not witness the arrest shared their upsetting experience when they found out about their parent’s incarceration,

“...I was in shock [when I found out]. I cried, and then, I ran to my room… I was pissed off… very much so because I knew that I could [have] at least said something at his court thing if they let me because I know my dad! And what they sentenced him for is stupid. …. I didn’t know [my dad] was arrested till almost a week later because my mom did not inform me about it! I wasn’t surprised by it because he’s been locked up [be]fore overnight… but there is just so much stuff we’ve gone through because there [were] so many different stories that I was like, ‘Is he in there because he actually did something or because people are lying?” …Parents and adults telling me that I ‘don’t know what’s going on’, [that] I ‘don’t even know my father’ and talking bad about him and everything.”

Another adolescent shared their frustration and stress in dealing with the uncertainty and the lack of information during this time,

**“**It was just stressful not really knowing what was going on, and there was… lots of false hope going on, and that was really stressful. And….it was the end of my 8^th^ grade, and I don’t know… I just obviously never anticipated for none of this to occur, so, that was probably the most stressful [thing about the arrest period]… not really knowing the status of everything…“…If we would’ve like known this would’ve been an actual outcome we could’ve literally prepared for everything because like even like [computer, online banking, email] passwords and stuff like that… all that stuff we just didn’t know we were going to have to deal with and stuff… and if we would have known even during the trial that we could’ve prepared so it wouldn’t be drastic such of change.” (20)

Caregivers also shared feelings of fear for stigmatization and negative treatment from others, or fear that the truth would cause more harm to the child. One caregiver relayed how she advised her child to not disclose maternal incarceration,

“Emotionally, not so good. I think that was because I told her not to broadcast [her] mom being [in] jail or prison…she was aware of ...[the] stigma involved with that, and it probably made her feel awkward. …The one [school] assignment that she had to do…[about her family]…She wrinkled it up and threw it across the room. She was extremely upset. And that was in kindergarten. That was a sad thing to see, that she would [get] that enraged. There had to be a lot [of] shame there I would guess.”

One adolescent also disclosed their experiences in concealing the truth out of fear of judgement from peers,

“I would have to lie to people [when peers] would ask me why my … mom [is] white or why [they] haven’t …met my real mom and [I] would just lie to them like… “she live in California and she only come around on the weekend” or something like that. I didn’t really want to tell them the honest truth because I was so young, I was scared of being judged …because I was already judged about my forehead and all this other stuff …”

Despite the experiences concealing the incarceration, recently released parents felt strongly that open and honest communication with the child would alleviate anxiety about the unknowns of the parent’s incarceration. Two of them shared the importance of honesty,

“Don’t lie, tell the truth… to the child. [Previously] I would say that I was at work or gone during my arrest and bond hearings and stuff. [Now] I would be honest I don’t care how old the child is, I would say just be honest.”

“Be truthful and be honest …that’s the only thing I could really say.”

### Limited Household and Community Resources to Help Family Deal with Consequences of Arrests

Upon arrest, families discussed difficulty in obtaining resources to better aid the children and navigating the custody process. Caregivers shared challenges specifically in taking over responsibilities of caring for the children, as they often lacked the resources to immediately do so. For example, one grandmother explained,

“…A crib, the walkers, and everything that comes along with it, the Bumbo seats, all that equipment that babies require…Diapers were huge. And the equipment that they need. Car seats, all that was not available to me because [the parent] was locked up and shipped out, and I couldn’t easily get into her home.”

Another grandmother expressed the need for financial resources to help provide for the child,

“Now, if it was just my income, I would’ve qualified for food stamps and Title 20, but [I was also living with my daughter and] my daughter’s child support put me over the income [threshold] to get help because [having her in the household made the income requirements] for a family of four [instead of a family of 3]… [Because the children’s mother died right before the dad was incarcerated] the kids’ social security death benefit from their mom disqualified them automatically for kinship care. And in order to get any kinship care, I was told I have to put them in children’s services, and then they give them back to me. And I’m not doing that! They’ve been through enough…so, it just makes it hard for those who really need the help to get it.”

The same caregiver described the legal challenges of accessing government support and custody of the child after the parent was detained,

"[I was] very disappointed, I guess, in [the] process [of] our legal system. Dynamics that are put in place for people who do not have any money…and are facing serious prison time and to me, any time in prison is serious, and then, to not feel like you are understanding the process.”

Other participants pointed out that residences were locked, and eligibility for supports was not allowed because the incarcerated parent had access, but extended kin did not. Instead, participants reported that assistance with understanding the availability of services and subsequently someone to assist them with the necessary services upon arrest would be valuable. One participant stated,

"Probably what would’ve been helpful at the time of arrest when I received the child would be a list of county resources. Should I continue to have possession of child [I found out later] that I could ask for assistance from whatever programs are available in the county. It took me a long time to find that. I was at a los*s*, I had no idea what to do [initially without knowing the resources available to us]. I was working, I had to find daycare, there … [were] a lot of things … Trying to keep my job, and take care of an infant, a tiny little infant. I was very concerned about the daycare, and the quality of the care, and the expense of the care. I was a grandma’s age! And I didn’t have young people around that I could tap into easily to be babysitters. And I was not old enough to retire, so I had to work. And I had no one else to help me.”

Another caregiver expressed notable differences across county resources and processes:

“… A County courts have not been as helpful as B County was. I don’t know if it was the caseload, I don’t know what the caseload is, but there’s a difference when it comes [to] A County anything, whether it’s welfare, or going through their courts, and dealing with B County. There’s a total difference. *A County just handles things and gets it done, whereas B County, you’re told no. or you have to do this or that, and then they’re still not helping you.*” (12)

Participants also described the need for emotional support to assist with processing the separation of the incarcerated family member. An adolescent shared,

“You just need people to talk to…you need someone to rant everything to and that will just soak it up and not judge you for what you’re saying…emotional support was very much needed… I only have my friends and stuff… I don’t really trust any adults or anything."

Caregivers acknowledged that they could use help to appropriately assist children across developmental stages in their understanding of their parent’s incarceration. Families recommended support services and developmentally appropriate handouts on how to explain the arrest and absence.

## Discussion

About a third of the participants in our study indicated that a child was present during the arrest of their parent – consistent with other evidence reporting similar percentages (22–40%) (Metcalfe et al., 2022). Our findings revealed difficult experiences for those present as family members disclosed the fear, confusion, and general uncertainty related to the arrest period of the parent. Upon arrest, family experiences were described as ‘traumatizing’ and ‘stigmatizing’ regardless of whether the child was present to observe the arrest. The effects of witnessing or concealing the arrest were influenced by officer treatment and fear. Results underscore the need for family-centered approaches and interventions upon arrest that may better support children and family members during this time. Recommendations for better transparency about this process and available resources (i.e., guidance sheets) were suggested by participants.

While some organizations and programs provide services to families affected by household incarceration (Geller et al., 2011), participants shared that these were not readily accessible for them, as supports may not be available in all jurisdictions. Additional infrastructure and support are needed for cross-sector collaboration, funding, and case management. The use of county liaisons or the assistance of social workers could direct families to custodial, legal, or financial services at the time of removal of the parent. The participants of this study particularly described how such a liaison could assist families on where to go, what to plan for, and how to communicate with their children about next steps. Schools are uniquely positioned to provide such direct and indirect student support services that can increase protective factors for children of incarcerated parents (Warren et al., 2019). To illustrate, a school counselor may offer support prior to and after visits with the newly arrested parent, facilitating a smoother experience and reducing any added emotional challenges for children. In line with our findings, previous evidence has further reiterated the need to reduce challenges that caregivers face at this time, as disruptions in the family unit make it more challenging for caregivers to take children to necessary healthcare provider appointments to receive preventative care (Khazanchi et al., 2019).

Families shared how limited social, economic, and community-based resources were available to help the family cope with the arrest. Substantial financial and legal barriers surrounding custody and kinship care were also noted. Participants expressed the need for improved transitional supports, such as standardized treatment from arresting officers with additional sensitivity training. Similar to the identified needs of the participants, a report from the 2009 National Conference of State Legislators (a non-partisan public officials association composed of sitting state legislators) encouraged agencies to develop protocols that outline next steps for families upon arrest (Christian, 2009). While the officer must consider many factors when conducting an arrest and maintain a priority for safety, there are available strategies that officers should be aware of to use when possible. The Child Development-Community Policing program is one collaborative model between law enforcement and mental health providers which involves training officers in child development and ensures that clinicians are present at the time of arrest (Berkowitz & Marans, 2000). The International Association of Chiefs of Police and Yale Child Study Center (2017) has also released a toolkit titled *Enhancing Police Responses to Children Exposed to Violence* which recommends several strategies that can assist officers in navigating an arrest while children are present. One technique for police to minimize these traumatic experiences is advising officers to treat the parent with respect during the arrest, and the skills to explain to the child in developmentally appropriate way what happened, why, and what will happen next. Additionally, officers can find examples of frequently asked questions by children and developmentally appropriate responses within this toolkit. These are important considerations for officers, as results from this study affirm evidence from Nolan (2003), who suggested that giving the child an opportunity to speak with the parent during an arrest can reduce traumatic symptoms. Other initiatives like the Support Team Assistance Response (STAR) program in Colorado allow medical professionals to respond to certain house calls related to mental health or substance use concerns and could possibly reduce parental arrest, child exposure to trauma, and poor officer and family interactions (Support Team Assistance Response, n.d). Therefore, better training and collaboration with health care organizations could aid in reducing the stress associated with witnessing a parent’s arrest.

### Study Limitations

Our study has several important limitations. First, we used a convenience-based sampling recruitment strategy and acknowledge that we may have missed important perspectives from families who had little access to the listservs, schools, and community organizations that we reached out to. Future research may continue to recruit from diverse populations who have experienced PI, as additional individuals may provide even deeper insights into the array of emotions experienced during this time. Despite our findings that reiterated a need for connection with an incarcerated parent, one previous mixed-methods study found that 31% of children participants reported negative feelings about their incarcerated parent, including resentment and anger (Shlafer & Poehlmann, 2010). Another previous study suggested that children who witness a parental arrest are significantly more likely to also witness and be victims of multiple types of violence in their homes (Phillips & Zhao, 2010). Professionals may thus ensure they continue to follow available guidelines when responding to certain situations, ensuring protection of the children (San Francisco Partnership for Incarcerated Parents, nd).

Second, most of the families used the term “jail” and “prison” interchangeably, and there are likely very important differences between experiences of families whose parent served time in prison, compared to jail. Unfortunately, we were unable to verify the context of the PI. Additional research is needed to understand contextual differences between these types of detainments and the effects of witnessing the arrest in relation to the age of the child. Moreover, participants completed the interview at different timeframes and at various ages after parental arrest. Future research is needed to explore developmental impacts of arrest on youth wellbeing in addition to examining potential differences in retrospective reporting among those who complete interviews closer to the time of arrest compared to not. Third, data collection was carried out in March- August of 2020 during the beginning of the COVID-19 pandemic. Additional household stress during this time and during recall of sensitive events could have impeded memory recall.

Despite these limitations, the results from this work provide insight and important context of the arrest period from the perspective of families impacted by incarceration. While the small sample size may limit generalizability of results, saturation of the data was met. The independence of the groups was also difficult to discern, yet we feel that this article provides a snapshot of experiences at the time of arrest that providers may reflect on when working with families impacted by parental incarceration to better support children’s health and well-being. Our paper not only highlights the available evidence regarding parental incarceration and its effects, but also underscores the limited previous research on family members during the time period of arrest, while prioritizing the voices of participants.

### Positionality Statement

We acknowledge the power and privilege of each research team member to conduct a study on families affected by incarceration. The research team was mostly based in Midwestern pediatric hospital institutions which aims to improve the health and well-being of children. We also acknowledge that perceptions, ideas, and understandings of incarceration are greatly governed by stereotypes and stigma. While all the authors have experienced familial incarceration in their lifetime (e.g., sibling, child, cousin, aunt, uncle, or grandparent, who served time in prison and/or jail etc.), only two of the authors have personally experienced an incarcerated parent during their childhood.

## Conclusion

This study provides insight into the experiences, challenges, and needs of families affected by PI during the time of parental arrest. Specifically, family experiences were described as traumatizing and stigmatizing, and were influenced by officer treatment. Due to the limited household- and community-based resources available for families, findings highlight the need for more transparency about available supports and programs that enhance family connection.

## Data Availability

N/A.
